# Remote Ischemic Preconditioning Does Not Affect the Release of Humoral Factors in Propofol-Anesthetized Cardiac Surgery Patients: A Secondary Analysis of the RIPHeart Study

**DOI:** 10.3390/ijms19041094

**Published:** 2018-04-05

**Authors:** Julia Ney, Katleen Hoffmann, Patrick Meybohm, Andreas Goetzenich, Sandra Kraemer, Carina Benstöm, Nina C. Weber, Johannes Bickenbach, Rolf Rossaint, Gernot Marx, Kai Zacharowski, Jürgen Bernhagen, Christian Stoppe

**Affiliations:** 1 Department of Anesthesiology, Medical Faculty, RWTH Aachen University, 52074 Aachen, Germany; rrossaint@ukaachen.de; 2 Department of Intensive Care Medicine, Medical Faculty, RWTH Aachen University, 52074 Aachen, Germany; caren.hoffmann@gmx.de (K.H.); cbenstoem@ukaachen.de (C.B.); jbickenbach@ukaachen.de (J.B.); gmarx@ukaachen.de (G.M.); 3 Departments of Anesthesiology, Intensive Care Medicine and Pain Therapy, University Hospital Frankfurt, 60590 Frankfurt, Germany; patrick.meybohm@kgu.de (P.M.); kai.zacharowski@kgu.de (K.Z.); 4 Department of Thoracic, Cardiac and Vascular Surgery, Medical Faculty, RWTH Aachen University, 52074 Aachen, Germany; andreas@goetzenich.net (A.G.); skraemer@ukaachen.de (S.K.); 5 Department of Anesthesiology & Laboratory of Experimental Intensive Care and Anesthesiology L.E.I.C.A, 1105 AZ Amsterdam, The Netherlands; N.C.Hauck@amc.uva.nl; 6 Vascular Biology, Institute for Stroke and Dementia Research, Ludwig-Maximilians-University of Munich Hospital, 80539 Munich, Germany; juergen.bernhagen@med.uni-muenchen.de

**Keywords:** remote ischemic preconditioning, molecular mechanisms, humoral factors, cardiac surgery, propofol anesthesia

## Abstract

In contrast to several smaller studies, which demonstrate that remote ischemic preconditioning (RIPC) reduces myocardial injury in patients that undergo cardiovascular surgery, the RIPHeart study failed to demonstrate beneficial effects of troponin release and clinical outcome in propofol-anesthetized cardiac surgery patients. Therefore, we addressed the potential biochemical mechanisms triggered by RIPC. This is a predefined prospective sub-analysis of the randomized and controlled RIPHeart study in cardiac surgery patients (*n* = 40) that was recently published. Blood samples were drawn from patients prior to surgery, after RIPC of four cycles of 5 min arm ischemia/5 min reperfusion (*n* = 19) and the sham (*n* = 21) procedure, after connection to cardiopulmonary bypass (CPB), at the end of surgery, 24 h postoperatively, and 48 h postoperatively for the measurement of troponin T, macrophage migration inhibitory factor (MIF), stromal cell-derived factor 1 (CXCL12), IL-6, CXCL8, and IL-10. After RIPC, right atrial tissue samples were taken for the measurement of extracellular-signal regulated kinase (ERK1/2), protein kinase B (AKT), Glycogen synthase kinase 3 (GSK-3β), protein kinase C (PKCε), and MIF content. RIPC did not significantly reduce the troponin release when compared with the sham procedure. MIF serum levels intraoperatively increased, peaking at intensive care unit (ICU) admission (with an increase of 48.04%, *p* = 0.164 in RIPC; and 69.64%, *p* = 0.023 over the baseline in the sham procedure), and decreased back to the baseline 24 h after surgery, with no differences between the groups. In the right atrial tissue, MIF content decreased after RIPC (1.040 ± 1.032 Arbitrary units [au] in RIPC vs. 2.028 ± 1.631 [au] in the sham procedure, *p* < 0.05). CXCL12 serum levels increased significantly over the baseline at the end of surgery, with no differences between the groups. ERK1/2, AKT, GSK-3β, and PKC_ɛ_ phosphorylation in the right atrial samples were no different between the groups. No difference was found in IL-6, CXCL8, and IL10 serum levels between the groups. In this cohort of cardiac surgery patients that received propofol anesthesia, we could not show a release of potential mediators of signaling, nor an effect on the inflammatory response, nor an activation of well-established protein kinases after RIPC. Based on these data, we cannot exclude that confounding factors, such as propofol, may have interfered with RIPC.

## 1. Introduction

Ischemic preconditioning has been widely introduced as it is the most powerful cardioprotective strategy to reduce myocardial infarct size. Since its first description by Murry et al. [1] in 1986, it has been tested in various in vitro and in vivo studies and has displayed impressive protective effects [2]. Not only can the stimulus originate from local repetitive ischemia/reperfusion stimuli at the heart, but also from those at remote organs or tissues—for example the upper limb, thus supporting the concept of remote ischemic preconditioning (RIPC) [3,4]. 

Early smaller clinical studies were stimulated by these promising findings, which confirmed that RIPC mediated the reduction of perioperative troponin release in patients after cardiac surgery [5,6]. Although promising, these results were initially limited to smaller patient populations and surrogate outcome measures of myocardial injury—rather than clinical outcomes—and fueled the need for adequately powered multicenter trials in order to test the clinical significance of RIPC. However, both of the recently published ERICCA [7] and RIPHeart [8] trials failed to demonstrate a reduction in postoperative troponin release and clinically significant effects of RIPC on postoperative outcomes in cardiac surgery patients. Although the underlying reasons remain largely speculative, it is currently being debated whether the RIPC stimulus was ineffective in these patients due to the use of the intravenous anesthetic propofol, which may have negatively affected and interfered with RIPC induced effects [9].

Besides the ambiguous effect of RIPC on clinical outcomes, the signal transduction of RIPC and the activation of intracellular signaling pathways is still largely unclear [10,11]—notably in humans. The macrophage migration inhibitory factor (MIF) and stromal cell-derived factor 1 (CXCL12) are potential ligands of G protein-coupled receptors (GPCRs) and share many characteristics that overlap with the mechanisms of ischemic preconditioning—including the activation of extracellular-signal regulated kinase (ERK1/2), protein kinase B (AKT), and protein kinase C (PKCε) [12,13,14]. Likewise, CXCL12 levels increase in response to hypoxia and activate the same kinases that are known to be involved in the reperfusion injury salvage kinase (RISK) pathway [15]. This led us to investigate whether MIF and CXCL12 levels increase after RIPC. 

Therefore, we have addressed potential biochemical mechanisms that are triggered by RIPC in the RIPHeart trial and hypothesized that they were influenced by the use of propofol.

## 2. Results

### 2.1. Patients

Of the patients that were screened in the Medical Faculty of the RWTH Aachen, 40 patients were randomly assigned to one of the intervention groups and consecutively enrolled in this secondary analysis and followed until the final analysis (Supplemental digital content, Figure S2). The included patients reflected a representative cohort of coronary artery bypass graft (CABG) patients from our center with typical co-morbidities and co-medications. Baseline and operative characteristics are shown in Table 1 and Table 2.

### 2.2. Cardioprotective Effects of RIPC—Perioperative Troponin T Release

Serum troponin T levels had increased significantly when compared with the baseline at any point in time (*p* < 0.01) postoperatively, but there was no significant difference between both of the treatment groups (Supplemental digital content, Figure S3). However, a trend towards lower troponin T levels in the RIPC group was observed.

### 2.3. Serum and Tissue Levels of MIF and Serum Levels of CXCL12 as Potential Mediators of RIPC

**Figure 1:**
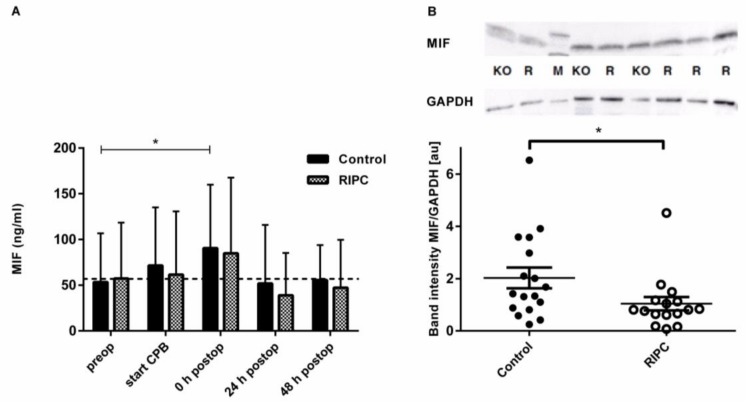
Macrophage migration inhibitory factor (MIF) concentration in serum and heart tissue samples. (**A**) Pre-, peri-, and postoperative concentrations of MIF in serum samples assessed by ELISA. (**B**) Densitometric analysis of MIF content in myocardial atrial tissue collected while connected to the cardiopulmonary bypass (CPB). MIF was determined via Western blotting and normalized to GAPDH. Data represents means ± standard deviation (SD); * *p* < 0.05 compared with the baseline.

Prior to surgery, both groups did not show significant differences with regard to MIF levels (53.4 ± 53.31 ng/mL in the control group vs. 57.35 ± 60.99 ng/mL in the RIPC group, *p* = 0.828) (Figure 1A and Supplementary Table S1). Immediately after RIPC, no significant changes in MIF levels had occurred either. After surgery, both of the groups demonstrated a rapid increase in MIF levels. In the control group, elevation of MIF values had reached statistical significance when compared with the baseline (90.59 ± 69.26 ng/mL, *p* = 0.023). In the RIPC group, MIF values were similarly high (84.9 ± 82.7 ng/mL, *p* = 0.164), however they did not reach statistical significance. After surgery, no significant differences between the RIPC and the control group were observed (*p* = 0.814). MIF levels had decreased close to the baseline values 24 and 48 h postoperatively. MIF concentrations in atrial tissue were significantly (*p* = 0.028) higher in the control group (2.03 ± 1.63 ng/mL) than in the RIPC group (1.04 ± 1.03 ng/mL) (Figure 1B).

**Figure 2:**
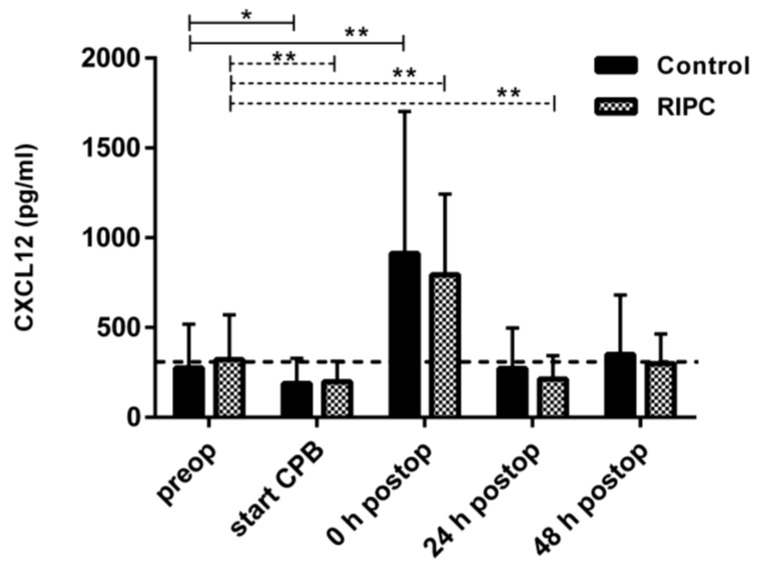
Secretion profile of proinflammatory cytokine, stromal cell-derived factor 1 (CXCL12), in control and remote ischemic preconditioning (RIPC) serum. Pre-, peri-, and postoperative concentrations of CXCL12 in serum samples assessed by ELISA. Data represents means ± SD; * *p* < 0.05; ** *p* < 0.01.

CXCL12 baseline levels did not differ between the groups (276.46 ± 243.45 pg/mL in the control group vs. 322.29 ± 247.90 pg/mL in the RIPC group, *p* = 0.5971). After the control or RIPC intervention, in both groups, CXCL12 had increased to a maximum after surgery. At the time of ICU admission, circulating CXCL12 levels were 2.46 times higher than the baseline levels in the RIPC group (793.85 ± 448.23 pg/mL, *p* = 0.001), and even 3.3 times higher in the control group (912.06 ± 789.812 pg/mL, *p* = 0.002) (Figure 2 and Supplementary Table S2). CXCL12 levels had decreased to preoperative values—24 and 48 h after ICU admission—demonstrating comparable perioperative CXCL12 kinetics as MIF, without significant differences between the control and the intervention group.

### 2.4. Serum Levels of IL-6, CXCL8, and IL-10

**Figure 3:**
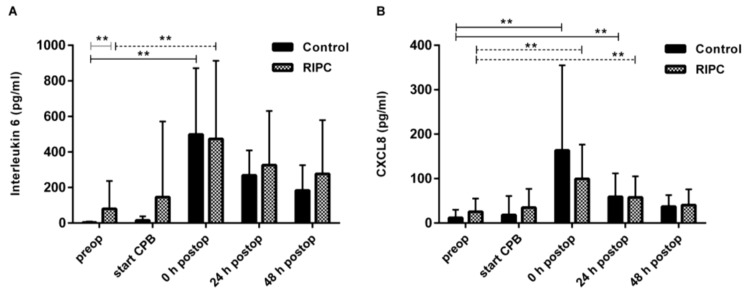
Secretion profile of proinflammatory cytokine IL-6 and CXCL8 in the control and RIPC serum. Pre-, peri-, and postoperative concentrations of (**A**) IL-6 and (**B**) CXCL8 in serum samples assessed by ELISA. Data represents means ± SD; ** *p* <0.01.

Postoperatively, interleukin levels had increased significantly over the baseline, without differences between the groups for the pro-inflammatory cytokine levels of IL-6 (Figure 3A) and CXCL8 (Figure 3B), or the anti-inflammatory cytokine IL-10. 

### 2.5. Western Blot Analysis of Protein Kinases ERK1/2, AKT, GSK-3β, and PKCε for Content and Phosphorylation

**Figure 4:**
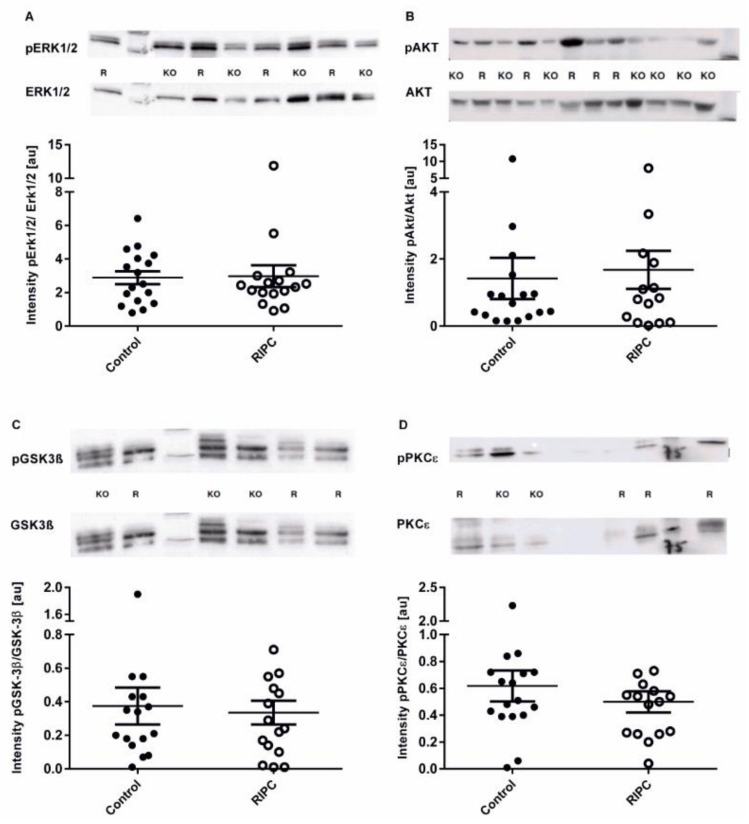
Extracellular-signal regulated kinase (ERK1/2), protein kinase B (AKT), GSK-3β, and protein kinase C (PKCε) activity on the RIPC in heart tissue. Densitometric analysis of ERK1/2, AKT, GSK-3β, and PKCε content in myocardial atrial tissue collected while connecting to the cardiopulmonary bypass (CPB). Relative phosphorylation of (**A**) ERK1/2, (**B**) AKT, (**C**) GSK-3β, and (**D**) PKCε were assessed by Western blotting and normalized to the unphosphorylated proteins. Data represents means ± SD.

To measure the potential effect of circulating mediators in the patients’ blood on the activation of protective kinases in the myocardium, we measured the activation of pro-survival kinases ERK1/2 and AKT. Both kinases—the RIPC and the control—showed a comparable activation 60 min after being treated (Figure 4). Additionally, no significant differences were observed in GSK-3β and PKCε activity in the myocardial tissue samples. Neither total nor phosphorylated protein content of AKT, ERK ½, PKC*ε*, and GSK-3β differed between groups.

## 3. Discussion

In the present study of our patient cohort undergoing RIPC under propofol anesthesia, we were unable to identify a humoral mediator or a protein kinase of the RISK and survivor activating factor enhancement (SAFE) pathway. Furthermore, in contrast to previous clinical studies [5,6], the perioperative troponin release did not significantly differ between the control and the active treatment group after cardiac surgery. Thus, the underlying reasons for the discrepancy of the positive results from previous, smaller clinical trials were called into question. 

Interestingly, in order to attenuate the cardioprotective effect of RIPC, several articles had suggested the use of the anesthetic agent propofol. Furthermore, Kottenberg et al [9] found a significant reduction of troponin I (by 50%) in the RIPC group—in CABG patients that received isoflurane anesthesia—when compared with the control group. Similarly, Bautin et al. [16] indicated a significant reduction of troponin I in the RIPC group—in patients undergoing aortic valve replacement—when using sevoflurane as anesthetic. Additionally, Zarbock et al. [17] demonstrated that when sevoflurane anesthesia was used, RIPC reduced the rate of kidney injury and the need for renal replacement therapy in high risk patients after cardiac surgery. Thus, supporting the hypothesis that volatile anesthetics do not influence the RIPC pathways and its organ protective effects. However, two meta-analyses reached opposing conclusions on the interference of propofol with RIPC and the protection by RIPC under volatile anesthesia [18,19]. 

These findings suggested that the anesthetic drug might possibly interfere with the signal transduction of RIPC. Hausenloy et al. [20] reported—from small animal studies—that on an intracellular level, RIPC resulted in an activation of the RISK pathway [21,22]—including the synthesis of several protein kinases (PI3K-AKT, ERK1/2, PKCε, and endothelial nitric oxide synthase). Skyschally et al. [23] reported—from studies involving pigs—that the SAFE pathway [24] involving the Janus kinase/Signal transducer and activator of Transcription protein (JAK/STAT) signalling activation was activated by RIPC [25,26]. Both pathways led to an inhibition of the mitochondrial permeability transition pore (mPTP) opening and connexin 43 activation. This resulted in reduced cell death by avoiding mitochondrial swelling [4]. Additionally, the SAFE pathway induced the transcription of pro-survival genes in the nucleus, which provided protective properties in the cardiomyocytes. To activate the RISK or the SAFE pathways, extracellular ligands were required to activate cell surface receptors [11]. Within the presently identified GPCRs, CXCR4 appeared to play an extraordinary role in RIPC. Yellon et al. [27] demonstrated that AMD3100—a highly specific inhibitor of the receptor CXCR4—abrogated the RIPC induced reduction of infarct size. Furthermore, CXCR4 is a known receptor for MIF [28] and CXCL12 [29,30]. The evidence suggests that MIF and CXCL12 are potential mediators of RIPC, as these cytokines are rapidly released in response to various stimuli—such as hypoxia [31]—and activate the organ-protective [32] kinases ERK1/2, AKT, and PKCε that overlap with the mechanisms of preconditioning. In the isolated cardiomyocytes of rats, MIF was released in response to a conditioning stimulus and resulted in signaling mechanisms typically observed in preconditioning [33]. Furthermore, in rats, CXCL12 serum levels increased after remote ischemic conditioning [27]. In our study, we were the first to attempt to translate the present experimental findings to humans. However, we were unable to demonstrate a significant difference in perioperatively measured MIF and CXCL12 levels between conditioned and non-conditioned patients. Moreover, we found that RIPC did not trigger the activation of the well-known prosurvival kinases—ERK1/2 and AKT—neither in the RIPC nor in the control group. Presently, it remains speculative whether propofol may have interfered with the RISK pathway by inhibition of released MIF and CXCL12. Thus, the inactivation of MIF, CXCL12, and other potential mediators might have interrupted the GPCR mediated signal cascade andits downstream effects. In rats, preconditioning with isoflurane resulted in a significant secretion of MIF in cardiomyocytes [32], thus further supporting the hypothesis that volatile anesthetics do not interfere with effects triggered by conditioning, which is in apparent contrast to propofol. 

In previous studies, RIPC also modulated the inflammatory response [34,35]. Therefore, we measured plasma IL-6, CXCL8, and IL-10. However, we did not confirm such effects in our study, further supporting the notion that intraoperative factors may have negatively affected the RIPC protective effects. Contrary to these results, Nederlof et al. [36] also found no effect of RIPC on mitochondrial hexokinase or on the inflammatory mediators in atrial tissue samples of sevoflurane-anesthetized cardiac surgery patients.

Moreover, it is currently being debated whether the RIPC stimulus algorithm has been ineffective. In fact, dose-finding studies in patients that undergo RIPC do not exist and the results of the two multicenter trials only indicate that an upper limb RIPC with four cycles of ischemia/5 min reperfusion, does not reduce myocardial injury. Bøtker et al. [37] indicated that the duration and number of the stimulus, determines the efficiency of RIPC with regard to post-ischemic hemodynamic recovery and infarct size, whereas one versus two limb ischemia has no significant influence. In particular, four to six cycles led to cardioprotection, whereas eight cycles did not provide additional effects [37]. Furthermore, there was no difference in outcome between the 2 min or 5 min ischemia cycle duration. However, 10 min ischemia seemed to abolish the cardioprotective effect [37]. For humans, such data do not exist.

Another intensively discussed topic is the potential protective effect of RIPC on organs other than the heart. In this context, Candilio et al. [38] described a similar cellular pathophysiology of ischemia reperfusion injury in several organs besides the heart—including the kidneys, the lungs, the intestine, the liver, and the brain. Thus, by upregulating circulating mediators and activating neural mechanisms [4], RIPC may provide protective properties in several organs. When comparing different organs, the brain appears to play an extraordinary role. A higher oxygen demand, reduced tolerance mechanisms, and lower protective antioxidant activities make this organ particularly sensitive to potentially harmful stimuli—in response to ischemic reperfusion injury—when compared with the heart, liver, kidneys, and lungs [39]. While smaller clinical trials exist [38,40], larger adequately designed multicenter trials are necessary to evaluate the potential effect of RIPC in other clinical settings, such as the hostile ischemia of the brain [41].

We acknowledge that our study had several limitations. Firstly, because this is a sub-analysis of a primary trial, we were not able to create a control group, receiving volatile anesthesia with or without RIPC. Consequently, we could only speculate that propofol anesthesia was the significant reason for RIPC to fail. Therefore, acceptable future studies are needed in order to compare the effect between RIPC and sevoflurane, and between RIPC and propofol. Another limitation of our study was that the depth of anesthesia and quantification of propofol usage had not been monitored. Further studies would be necessary in order to evaluate a possible correlation. Furthermore, as previously discussed, other possible confounding factors would have to be addressed cautiously and separately when interpreting the neutral effects that were observed in the RIPHeart study. For example, healthier subjects (stratified by EuroSCORE <5) demonstrated higher MIF levels when they were treated with RIPC. Another possible confounding factor, which may have interfered with the RIPC induced effect, was a longer cardiopulmonary bypass (143 vs. 120 min, *p* = 0.063) and a prolonged aortic cross-clamping time (99 vs. 79 min, *p* = 0.083) in patients that underwent RIPC in our cohort, even though the difference did not reach statistical significance. Although the actual data did not provide evidence that potential confounding factors diminished the effect of RIPC [42], acceptable future studies would have to systematically analyze whether comorbidities as well as commonly used medications of patients with cardiovascular diseases might interfere with RIPC-induced effects. Lastly, in the present analysis, we only focused on a few potential mediators of RIPC, however, increasing evidence suggests that multiple markers are involved in the mechanisms of RIPC. 

## 4. Methods and Materials

This study was approved in February 2010 by the University’s Institutional Review Board and the local ethics committee Aachen (EK 145/11, 15 March 2013), and written informed consent was obtained from all of the subjects that were participating in the trial. All of the patients that were included in this secondary analysis were part of the RIPHeart trial. The RIPHeart trial was registered, prior to patient enrollment, at clinicaltrials.gov (NCT01067703; Principal investigator: Patrick Meybohm; Date of registration: 11 February 2010) [8]. While the RIPHeart study investigated the influence of RIPC on a composite end point (death from any cause, myocardial infarction, new stroke, and acute renal failure), this predefined sub-study aimed to evaluate the role of potential mediators of RIPC in patients undergoing cardiac surgery. The additional intervention—in order to collect pre-, intra-, and postoperative serum and intraoperative heart tissue samples from the right atrium—was approved by the appropriate Institutional Review Board and the local ethics committee Aachen (EK 145/11), and written informed consent was obtained from all of the subjects.

### 4.1. Study Design, Patients, Randomization

There were 40 patients undergoing elective cardiac surgery that were enrolled from the prospective, randomized, double blinded, multicenter RIPHeart trial. Patients that were 18 years of age or older, that were scheduled for elective cardiovascular surgery requiring cardiopulmonary bypass (CPB) and that provided written informed consent, were eligible for enrollment. Key exclusion criteria were related to specific surgical procedures (e.g., off-pump heart surgery or urgent surgery) and severe organ dysfunction (e.g., ejection fraction <30% or severe renal failure). Patients were randomly assigned to the intervention or control group by the Clinical Trial Centre Leipzig (Zentrum für Klinische Studien Leipzig, 04107 Leipzig, Germany), using an internet-based randomization tool. The details have previously been published [8].

### 4.2. Surgical Procedure, Anesthesia, Intervention, Data Collection, and Blinding

After arriving at the operating room, anesthesia was induced—using the intravenous anesthetic propofol, the muscle relaxant rocuronium, and sufentanil—and maintained using total intravenous anesthesia with propofol. After patients were asleep and an arterial line had been placed, the first blood sampling was performed. Then, the RIPC procedure was started, which consisted of four cycles of upper-limb ischemia—5 min of blood-pressure cuff inflation to ≥200 mm Hg (but at least 15 mm Hg higher than the patient’s actual systolic arterial pressure) followed by 5 min of cuff deflation—on the intervention group, and four cycles of cuff inflation and deflation on a dummy arm in the control group (the sham intervention). While the staff—who carried out the RIPC protocol—were aware of the study-group allocation, the patients, anesthetists, and surgeons were blinded to the intervention. Furthermore, all of the other team members involved in the postoperative care and in documenting and analyzing the data were unaware of study-group assignments. After the completion of the RIPC protocol, the cardiac surgery was started. The right atrial tissue samples and the second blood sample were collected after the right atrium had been opened. After surgery, the sedated and intubated patients were transferred to the intensive care unit (ICU), where the third blood sample was taken. Additionally, 24 and 48 h after ICU admission, blood samples were collected (Supplemental digital content, Figure S1).

### 4.3. Laboratory Assessment 

All of the blood samples were collected in Serum-Monovettes (Sarstedt AG & Co, Nümbrecht, Germany). After the blood sampling, all of the samples were immediately centrifuged for 10 min at 3000 rpm and the supernatant was stored in Eppendorf tubes at −20 °C. MIF, IL-6, CXCL8, IL-10, CXCL12, and troponin T were analyzed using enzyme-linked immunosorbent assay (ELISA)-kits from R&D Systems (Minneapolis, MN, USA) according to the manufacturer’s instructions. Serum samples were diluted prior to assay (MIF–ELISA 1:400; IL-6, CXCL8, IL-10, and CXCL12 1:5) and double determination was carried out. 

The content and phosphorylation of ERK1/2, AKT, PKCε, GSK-3β, and the content of MIF in right atrial tissue samples were determined using SDS–PAGE and Western blot analysis. Heart tissue samples were shock–frozen in liquid nitrogen and stored at −20 °C until further analysis. For protein isolation, 30–50 mg of the tissue samples were mixed with 800 μL radioimmunoprecipitation assay (RIPA) complete buffer—containing 150 mM NaCl, 1% NP-40, 1% Natrium-Deoxycholate, 25 mM TrisHCL (pH 7.6), a phosphatase inhibitor cocktail (PhosSTOP; Roche, Mannheim, Germany), and a protease inhibitor cocktail (Complete; Roche, Mannheim, Germany)—and lysed with the Potter-Elvehjem homogenizer. Protein concentration was measured using a colorimetric assay (DC Protein Assay; Bio-Rad, Munich, Germany). For SDS–PAGE, 20 µg of protein was boiled in a LDS buffer (containing 100 mM Dithiothreitol) for 5 min at 95 °C. Samples were then separated via SDS–PAGE and subjected to semi-dry Western blotting. In order to detect proteins, blots were blocked for 60 min at room temperature with 5% BSA in T-TBS (TBS containing 0.05% Tween 20) and incubated with the primary antibody overnight at 4 °C. Blots were then washed with T-TBS and incubated with the appropriate secondary antibody. The following antibodies were used: anti-hMIF (Ka565; from Professor Bernhagen; dilution of 1:1000) in relation to GAPDH (Cell Signaling; Danvers, MA, USA; dilution of 1:1000), as well as the phosphorylated versus unphosphorylated form of AKT, ERK1/2, PKCε, and GSK-3β (Cell Signaling; Danvers, MA, USA; dilution of 1:1000). Band intensities were visualized with the enhanced chemiluminescence system (ECL) and the ChemieDoc MP System (BioRad, Munich, Germany) imaging system and analyzed using the program Image Lab (BioRad, Munich, Germany).

### 4.4. Statistical Analysis

All of the statistical analyses were performed using IBM SPSS Version 20 (IBM Corporation, Armonk, NY, USA). Figures were composed using GraphPad PRISM^®^ Version 6 (GraphPad Software Inc., La Jolla, CA, USA). Normal distribution was verified via the Shapiro–Wilk test. Given the exploratory nature of the present study, changes of normally distributed parameters were tested with unpaired *t*-tests, while the Mann–Whitney U test was used for skewed data. The differences were considered statistically significant at *p*-values of ≤0.05. In addition to mean values, their standard deviation (SD) was measured and reported as ± SD. 

## 5. Conclusions

In this cohort of patients that underwent cardiac surgery, RIPC did not exert cardioprotective effects. We also found no differences between the RIPC and the control in circulating inflammatory plasma factors and right atrial protein kinase activation. Confounding factors, such as propofol, may have interfered with the RIPC protective signaling.

## Figures and Tables

**Table 1:** Baseline characteristics.

Characteristics	Control (*n* = 21)	RIPC (*n* = 19)	*p*-Value
**Demographic data**			
Age (years)	67 (45–84) *	67 (51–80)	0.860
Male (N, %)	12 (57)	14 (74)	0.287
Height (cm)	167 (145–185) *	173 (146–190)	0.047
Weight (kg)	77 (57–103) *	85 (58–117)	0.158
Logistic EuroSCORE *	2.2 (0.6–5.5)	1.7 (0.5–6.2)	0.892
**Comorbidities, No. (%)**			
Coronary heart disease	17 (76)	12 (63)	0.221
Hypertension	17 (76)	16 (84)	0.545
COPD	1 (5)	2 (11)	0.514
Diabetes	8 (38)	5 (26)	0.443
Stroke in the past	6 (29)	3 (16)	0.394
**Medication, No. (%)**			
Beta-blockers	17 (76)	14 (74)	0.600
ACE/AT1 inhibitors	14 (67)	14 (74)	0.645
Statins	18 (86)	16 (84)	0.912
Ca^2+^-channel blockers	6 (29)	2 (11)	0.165
Aspirin	17 (81)	16 (84)	0.805
Insulin/Metformin	5 (24)	2 (11)	0.545
Diuretics	11 (52)	9 (47)	0.230

* Median and range; COPD = Chronic obstructive pulmonary disease; ACE = Angiotensin Converting Enzyme; AT1 = angiotensin II type 1; RIPC = Remote ischemic preconditioning.

**Table 2:** Operative characteristics.

Characteristics?	Control Group (*n* = 21)	RIPC Group (*n* = 19)	*p*-Value
**Procedure, No. (%)**			
CABG only	11 (52)	9 (47)	0.759
Valve only	1 (5)	2 (11)	0.502
Combined	9 (43)	8 (42)	0.962
**Intraoperative times, Median (IQR), min**			
Time until CPB circulation	105.48 (65–145)	106.84 (80–150)	0.871
CPB in total	120 (30–232)	143 (67–247)	0.063
Aortic cross-clamping	79 (15–146)	99 (50–166)	0.083
Reperfusion on CPB	32 (6–77)	38 (9–77)	0.163

CPB = cardiopulmonary bypass; CABG = coronary artery bypass grafting.

## Supplementary Materials

Supplementary materials can be found at http://www.mdpi.com/1422-0067/19/4/1094/s1.
