# Supplementary material for: Remote Ischemic Preconditioning Does Not Affect the Release of Humoral Factors in Propofol-Anesthetized Cardiac Surgery Patients: A Secondary Analysis of the RIPHeart Study
Source: Int J Mol Sci. 2018 Apr 5;19(4):1094. doi: 10.3390/ijms19041094 (PMC5979505; doi:10.3390/ijms19041094)
Supplement: Supplementary file 1 [file ijms-19-01094-s001.pdf]

Supplemental digital content

Figure S1 Experimental procedure

Figure S2 Flow chart of study participants

Figure S3 Troponin T levels after surgery

Table S1 Pre- and postoperative MIF concentration in serum samples

Table S2 Pre- and postoperative CXCL12 concentration in serum samples

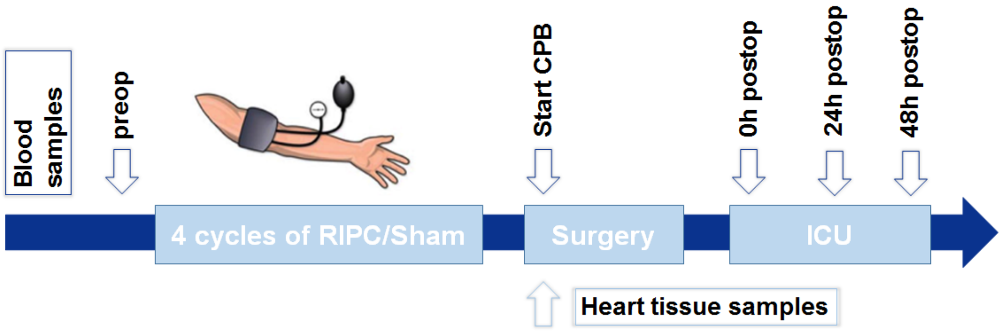

Figure S1 Experimental procedure. Blood samples were drawn prior to surgery, after RIPC by 4 cycles of 5min arm ischemia/5 min reperfusion (n=19) and sham (n=21) procedure, after connection to cardiopulmonary bypass (CPB), at the end of surgery, 24h postoperatively and 48h after surgery.

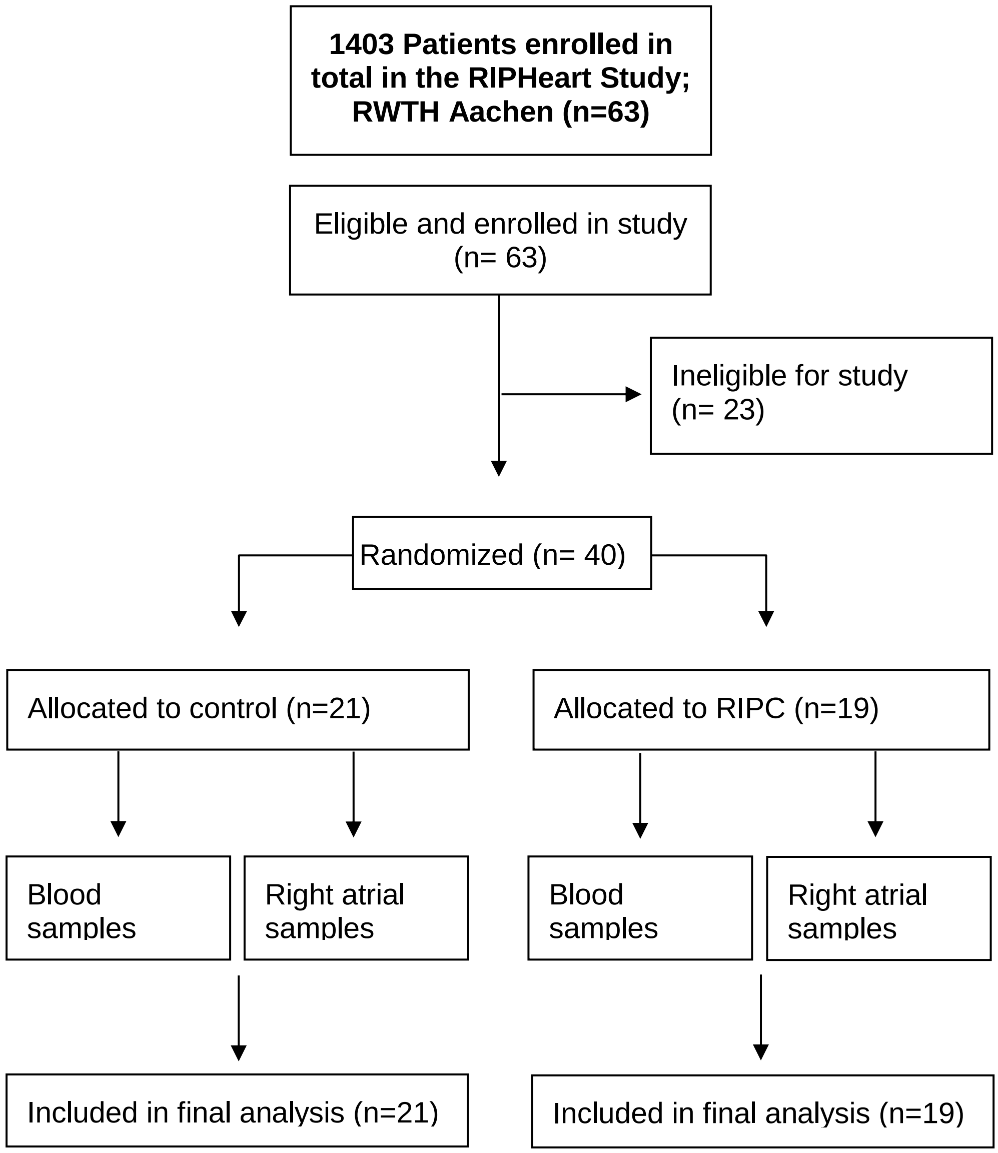

Figure S2. Flow chart of study participants.

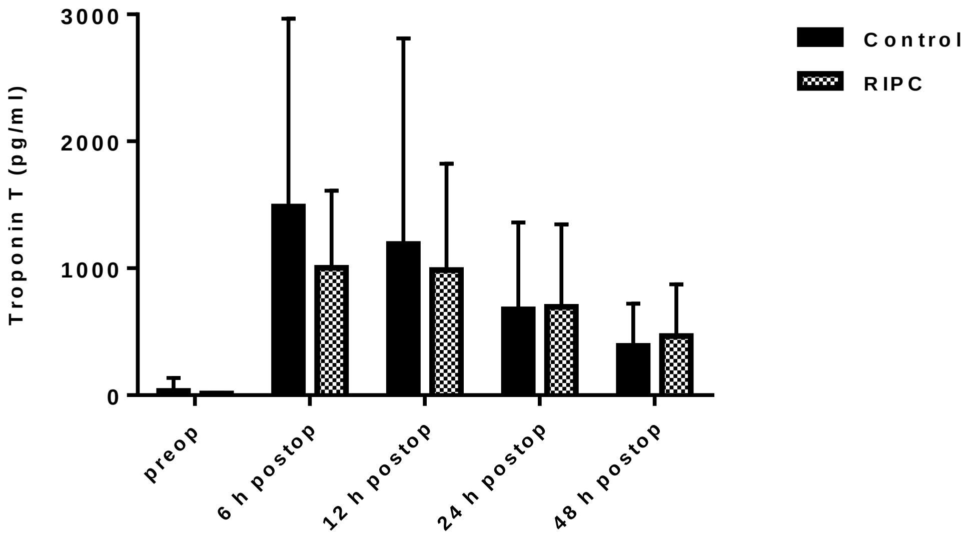

Figure S3. Troponin T levels after surgery. Troponin T levels were measured before surgery (Pre-OP) and 6h, 12h, 24h, 48h after cardiac surgery. Data represents means+/-SD.

Table S1 MIF data.

	Control group (n=21)		RIPC group (n=19)		
MIF (ng/ml)	Mean ± SD	p-value compared to baseline	Mean ± SD	p-value compared to baseline	p-value comparing groups
A (before surgery)	53.4 ±53.31	1	57.35 ±60.99	1	0.828
X (after RIPC, before HLM)	71.58 ±63.51	0.255	61.62 ±69.15	0.802	0.665
B (ICU admission)	90.59 ±69.26	0.023	84.9 ±82.7	0.164	0.814
D (24h after surgery)	52 ±63.98	0.921	38.86±46.4	0.10	0.466
E (48h after surgery)	55.54 ±38,46	0.812	47.29 ±52.28	0.426	0.587

Table S2 CXCL12 data.

	Control group (n=21)		RIPC group (n=19)		
CXCL12 (pg/ml)	Mean ± SD	p-value compared to baseline	Mean ± SD	p-value compared to baseline	p-value comparing groups
A (before surgery)	276.46±243.45	1	322.29±247.89	1	0.597
X (after RIPC, before HLM)	188.60±140.38	0.029	198.88±113.51	0.001	0.827
B (ICU admission)	912.05±789.81	0.002	793.85±448.23	0.001	0.607
D (24h after surgery)	271.94±226.32	0.934	214.71±128.65	0.008	0.405
E (48h after surgery)	351.08±329.60	0.364	299.34±164.49	0.597	0.587
